# Clinical efficacy and safety of omalizumab in conventional treatment‐resistant vernal keratoconjunctivitis: Our experience and literature review

**DOI:** 10.1002/iid3.384

**Published:** 2021-01-12

**Authors:** Sara Manti, Giuseppe Fabio Parisi, Maria Papale, Gian Luigi Marseglia, Amelia Licari, Salvatore Leonardi

**Affiliations:** ^1^ Pediatric Respiratory Unit, Department of Clinical and Experimental Medicine University of Catania Catania Italy; ^2^ Department of Pediatrics, Fondazione IRCCS Policlinico San Matteo University of Pavia Pavia Italy

## 1 INTRODUCTION

Vernal keratoconjunctivitis (VKC) is a severe ocular disease characterized by recurring acute and/or chronic corneal‐conjunctival inflammation leading to visual sequelae.^1^ Since no treatments are universally effective in the management of VKC, novel therapies are currently under investigation, including anti‐immunoglobulin E (IgE). Although the efficacy and safety of omalizumab have been assessed in the therapeutic management of IgE‐mediated disorders^2^, ^3^, ^4^, ^5^, ^6^, ^7^; literature data on the use of omalizumab in VKC are very sparse.^8^, ^9^, ^10^, ^11^, ^12^, ^13^ Herein, we reported the use of omalizumab in treating conventional treatment‐resistant VKC unresponsive in two children. Finally, an overview of the available literature data is also reported.

## 2 CASE REPORTS

### 2.1 Patient 1

A 12‐year‐old boy reported severe allergic asthma since preschool age with house dust mite and grass pollen allergy detected by skin prick test (SPT) (dermatophagoides pteronyssinus 7 mm, dermatophagoides farinae 6 mm and grass pollen 5 mm) and specific IgE levels (121, 83 and 77 IU/ml, respectively), with high total IgE (2004 IU/ml) levels and normal eosinophil count (228/mmc). The spirometry showed a forced expiratory volume in 1 s (FEV1) of 57% and a forced vital capacity (FVC) of 82% of predicted. His disease control was low, despite daily high‐dose inhaled corticosteroid (ICS) plus inhaled long‐acting beta‐agonist (LABA) (fluticasone 1000 mcg/die + salmeterol 100 mcg/die) and leukotriene receptor antagonist (LTRA) (montelukast 10 mg/die).

From the age of 8 years, he also experienced painful gritty sensation of both eyes, photophobia, and stringy mucous discharge that worsened in the morning. Ocular manifestations were initially mild and were mistaken by the ophthalmologist for allergic conjunctivitis. With the worsening of the clinical picture, the subsequent ophthalmological evaluation showed large papillae of different shapes and sizes with Trantas' dots and infiltrates on the limbus. Superficial keratitis and chemosis were observed bilaterally. Because of the clinical symptoms and the physical examination, a clinical diagnosis of VKC was made. Unfortunately, he reported conventional treatment‐resistant VKC including topical antihistamines, mast‐cell stabilizers, corticosteroids (CS), and cyclosporine, and partially and temporarily responsive to treatment with systemic CS.

Treatment with subcutaneous omalizumab was started with the administration of 450 mg every 14 days according to the standards approved for patients with asthma with reference to the maximum dosages allowed based on weight and off‐label since the total IgE values exceeded the threshold value of 1500 IU/ml.^2^ Omalizumab was administered for nine consecutive months, from November 2018 to August 2019, and he is currently under treatment. For 3 months, we administered omalizumab as an add‐on therapy; subsequently, we progressively carried out therapy with ICS + LABA and LTRA.

At every visit, the treatment response in VKC was assessed both by ophthalmologic examination and using a visual analog scale evaluating the changes in ocular symptoms, such as burning and/or itching, redness, lachrymation, and photophobia. Signs and symptoms were also classified using disease severity scales, as previously described. Topical antihistamines was permitted as rescue drugs. Adverse events data were also collected.

**Figure 1:**
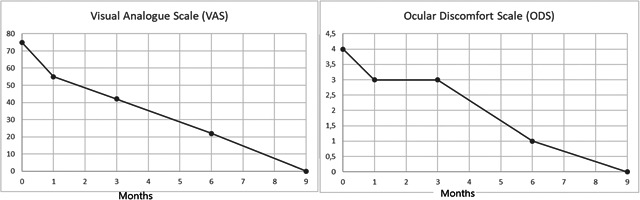
Changes in the visual analog scale (VAS) and in ocular discomfort score (ODS) for Patient 1

During this treatment period, he reported significant improvement in clinical symptoms, in severity disease, in need for rescue, and in ocular examination findings: eye redness and cobblestone papillae disappeared (Figure 1). Any adverse effect during the omalizumab treatment period was reported. Improvement in asthma was also identified both in terms of improvement of respiratory function (FEV1 99%, FVC 115%) and in terms of reduction in drug consumption (no therapy needed other than omalizumab).

### 2.2 Patient 2

**Figure 2:**
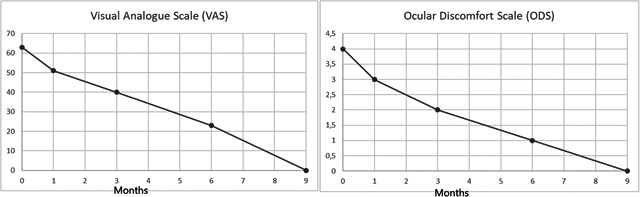
Changes in the VAS and in ODS for Patient 2. OSD, ocular discomfort score; VAS, visual analog scale

10‐year‐old boy with severe allergic asthma, grass pollen and parietaria allergy detected by SPT (grass pollen 9 mm and parietaria pollen 6 mm) and specific IgE levels (79.2 and 19.6 IU/ml, respectively), with high total IgE (2498 IU/ml) levels and increased eosinophil count (1493/mmc). The spirometry recorded FEV1 75% and FVC 85%. Low disease control, despite inhaled fluticasone 500 mcg/die + LABA 100 mcg/die and montelukast. From the age of 6 years, ocular disorders similar to those of patient 1 with associated periorbital dermatitis. After diagnosis of VKC, begin treatment with topical antihistamines, mast‐cell stabilizers, CS, and cyclosporine, with no satisfactory clinical improvement. As well as Patient 1, we started treatment with omalizumab (600 mg every 14 days), reporting significant improvement in clinical symptoms, in severity disease, in need for rescue therapies, and in ocular examination findings (Figure 2). No adverse effects were reported and improvement in asthma was also identified (FEV1 95%, FVC 114%).

## 3 DISCUSSION

VKC is a severe ocular disease affecting especially the pediatric population.^1^ The mechanisms governing the pathogenesis of VKC are still under investigation. Similarly to other disorders, VKC is featured by immediate and delayed hypersensitivity reactions, both IgE and non‐IgE mediated.^1^ Following the allergen binding to IgE, mast cells and basophils release histamine and proinflammatory cytokines. Accordingly, higher histamine concentrations, plasma cells, mast cells, macrophages, eosinophils, basophils, and fibroblasts are detecteed in VKC patients than control. Elevated Th2‐mediated cytokines, such as interleukin‐3 (IL‐3), IL‐4, and IL‐5, are detected in tears of patients affected by VKC.^1^ Increased IL‐2, interferon‐gamma, and tumor necrosis factor‐beta levels are the expression of Th1 immune response. Finally, neurotrophic factors, chemokines, mast cell‐released proteases.

Moreover, an impaired collagen synthesis further contributes to tissue damage.^1^ Several treatments aim at interrupting and/or preventing the proinflammatory immune response, such as H1 receptor antagonists and mast cell stabilizers and CS. Topical immunosuppressive agents, including cyclosporine A and tacrolimus, have been investigated as treatment for moderate to severe VKC. Nevertheless, numerous patients are unresponsive to the above‐mentioned treatment and/or may need treatment for longer period.^1^

Moreover, documentation on the treatments licensed for VKC treatment did not show a good safety profile, and long‐term outcomes are the significant limitations of all the therapies mentioned above.^1^ Considering the Th2‐mediated mechanisms underlying the pathogenesis of VKC, and aiming to identify a better therapeutic weapon against the disease, the efficacy of omalizumab has been recently disputed in the treatment of VKC both in adult and children. Omalizumab is a humanized monoclonal antibody that causes a reduction in IgE levels by inhibiting the binding of these immunoglobulins to the high‐affinity receptor for the Fc region of IgE (FcεRI) of mast cells and other inflammatory cells. This action inhibits the synthesis of mediators: cytokines and chemokines from inflammatory cells and reduces the presentation of antigens by dendritic cells.^2^ It is possible that omalizumab reduces inflammatory cells at conjunctiva since it is effective in “Th2‐high” asthma endotype.^14^

To date, in child and adolescent populations, omalizumab was shown to be effective treatment in VKC (Table 1),^8^, ^9^, ^10^, ^11^, ^12^, ^13^ except for a child reported in the series of Doan et al.^12^ Authors hypothesized that the not atopic status and a more severe phenotype of disease could negatively affect the efficacy of the omalizumab.^12^ Other authors did not confirm these findings.^8^ In the studies where it is reported, the onset of benefits was almost immediate, that is, within a few months from the beginning of treatment with omalizumab.^8^, ^9^, ^10^, ^11^, ^12^, ^13^

**Table 1:** Demographic and clinical features and outcomes in pediatric patients with VKC treated with omalizumab

**Authors**	**Design (*n* = number)**	**Age (years)**	**Comor‐bidities**	**SPTs**	**IgE**	**Clinical severity score pre‐O**	**Rescue medication**	**Omalizumab dosage**	**DTO**	**Relapse after treatment**	**Clinical severity score post‐O**	**AEs**
Sanchez et al.	Case study (1)	5	Asthma Rhinitis Eczema	Dust mite	340	NA	Not needed	300 mcg/2 weeks	6 Weeks	None	NA	None
Occasi et al.	Case study (4)	6	Eczema	NA	NS	NS	Not needed	225 mcg/2 weeks	6 Months	None		NS
		8	None					300 mcg/4 weeks				
		11	Rhinitis					225 mcg/4 weeks				
		9	Rhinitis					225 mcg/4 weeks				
Doan et al.	Retrospective study (4)	13	A + R	Grass pollens, fagaceaealternaria, cat dander, cow milk, eggs	146	4	NS	600 mcg/2 weeks	16 Months	None	3	NS
		10	A + R	Grass pollens, dust mites, alternaria, nuts	1655	4		600 mcg/2 weeks	33 Months	None	3	NS
		7	A + R	Grass pollens, birch, peanuts, eggs, kiwi	8000	4		450 mcg/2 weeks	42 Months	None	3	NS
		7	Asthma	None	141	4		600 mcg/2 weeks	6 Months	No response to treatment	4	NA
Heffler et al.	Case study (2)	7	None	None	NS	NS	NS NS	300 mcg/4 weeks	6 Months	NA	Disappearance of symptoms	None
		8	Eczema	None	NS	NS		600 mcg/4 weeks	6 Months	NA	Partial reduction of symptoms	None
de Klerk et al.	Case study (1)	12	Asthma, eczema, rhinitis	NS	NS	NA	Not needed	300 mcg/4 weeks	18 Months	None	NA	None
Current paper	Case study (2)	12	Asthma	Dp, Df, grass pollen	2004	4	Not needed	450 mcg/2 weeks	9 Months	None	0	None
		10	Asthma	Grass pollen parietaria pollen	2498	4	Not needed	600 mcg/2 weeks	9 Months	None	0	None

Abbreviations: AE, adverse effect; A + R: asthma + rhinitis; Dp, dermatophagoides pteronyssinus; Df, dermatophagoides farinae; DTO, duration of treatment with omalizumab; NA, not available; NS, not specified; SPT, skin prick test; VAS, visual analogic scale.

Currently, dosage instructions for omalizumab are lacking. Except for one adult patient receiving a single dose of omalizumab therapy,^13^ other studies have reported doses and duration of treatment schedules of omalizumab calculated according to the standards approved for patients with asthma.^8^, ^9^, ^10^, ^11^, ^12^, ^13^

Wide variability also exists in the treatment duration, and 42 months are reported as the most prolonged duration of treatment.^12^ Accordingly, if a single dose of omalizumab could increase patient compliance and decrease health care costs, the inconvenience of an incomplete efficacy of treatment and poor control of the disease can not be excluded. However, no relapses have been observed after treatment suspension.^8^, ^10^, ^11^

Literature and real‐life data on the long‐lasting effects of omalizumab in the treatment of VKC are lacking. Authors reported that the clinical effects were still present after 18 months of treatment with omalizumab.^10^, ^11^

Moreover, if some patients did not require different medications in association with omalizumab treatment, other subjects needed for topical steroids but at a lower dosage.^12^

## 4 CONCLUSION

Our contribution confirms the efficacy and safety of omalizumab therapy and justifies the urgent need to start large prospective randomized controlled trials on the omalizumab use to be licensed in treating of severe and conventional treatment‐resistant VKC.

## CONFLICT OF INTERESTS

The authors declare that there are no conflict of interests.

## INFORMED CONSENT STATEMENT

A written informed consent was obtained from the parents and informed assent from the patients.

## AUTHOR CONTRIBUTIONS

Salvatore Leonardi and Gian Luigi Marseglia designed the study. Sara Mant, Giuseppe Fabio Parisi, and Maria Papale enrolled the children and contributed to the data collection. Sara Mant and Giuseppe Fabio Parisi contribute to the follow‐up. Sara Mant wrote the initial draft of the manuscript. Salvatore Leonardi, Gian Luigi Marseglia, and Amelia Licari performed a critical revision of the manuscript and offered precious technical advice on how the study might be improved. All authors provided substantial contributions to the conception or design of the work, or the acquisition, analysis, or interpretation of data for the paper, revised the manuscript for important intellectual content, approved the final version, and agreed to be accountable for all aspects of the work.

## Data Availability

The datasets used and/or analyzed during the current study are available from the corresponding author on reasonable request.
